# Genetic structure of *Anopheles gambiae s.s* populations following the use of insecticides on several consecutive years in southern Benin

**DOI:** 10.1186/s41182-019-0151-z

**Published:** 2019-04-11

**Authors:** Arsène Jacques Y. H. Fassinou, Come Z. Koukpo, Razaki A. Ossè, Fiacre R. Agossa, Benoit S. Assogba, Aboubakar Sidick, Wilfrid T. Sèwadé, Martin C. Akogbéto, Michel Sèzonlin

**Affiliations:** 1 Cotonou Entomological Research Center (CREC), Cotonou, Benin; 2 Laboratory of Evolution, Biodiversity of Arthropods and Sanitation, FAST – UAC, Abomey-Calavi, Benin; 3 Graduate School of Life Sciences and Earth, FAST – UAC, Abomey-Calavi, Benin; 4 National University of Agriculture (UNA), Porto-Novo, Benin; 5 Medical Research Council Unit the Gambia at the London School of Hygiene & Tropical, Serrekunda, Gambia

**Keywords:** *Anopheles gambiae s.l.*, Genetic structure, Malaria, Resistance, Benin

## Abstract

**Background:**

Several studies have reported the strong resistance of *Anopheles gambiae s.l.* complex species to pyrethroids. The voltage-dependent sodium channel (*Vgsc*) gene is the main target of pyrethroids and DDT. In Benin, the frequency of the resistant allele (*L1014F*) of this gene varies along the north-south transect. Monitoring the evolution of resistance is necessary to better appreciate the genetic structure of vector populations in localities subject to the intensive use of chemicals associated with other control initiatives. The purpose of this study was to map the distribution of pyrethroid insecticide resistance alleles of the *Kdr* gene in malaria vectors in different regions and ecological facies in order to identify the evolutionary forces that might be the basis of anopheline population dynamics.

**Methods:**

The characterization of *Anopheles gambiae s.l.* populations and resistance mechanisms were performed using adult mosquitoes obtained from larvae collected in the four agroecological zones in southern Benin. Genomic DNA extraction was performed on whole mosquitoes.

The extracted genomic DNA from them were used for the molecular identification of species in *Anopheles gambiae s.l.* complex and the identification of genotypes related to pyrethroid resistance as the *Kdr* gene amino acid position 1014 in sodium channel. Molecular speciation and genotyping of *Kdr* resistant alleles (1014) were done using PCR.

Genepop software version 4.2 was used to calculate allelic and genotypic frequencies in each agroecological zone. The *p* value of the allelic frequency was determined using the binomial test function in R version 3.3.3. The Hardy-Weinberg equilibrium was checked for each population with Genetics software version 1.3.8.1. The observed heterozygosity and the expected heterozygosity as well as the fixation index and genetic differentiation index within and between populations were calculated using Genepop software version 4.2.

**Results:**

During the study period, *Anopheles coluzzii* was the major species in all agroecological zones while *Anopheles gambiae* was scarcely represented. Regardless of the species, resistant homozygote individuals (*L1014F/L1014F*) were dominant in all agroecological zones, showing a strong selection of the resistant allele (*L1014F*). All populations showed a deficit of heterozygosity. No genetic differentiation was observed between the different populations of the two species. For *Anopheles coluzzii*, there was a small differentiation among the populations of the central cotton and bar-lands zones. The genetic differentiation was modest among the population of the fisheries zone (*Fst* = 0.1295). The genetic differentiation was very high in the population of *Anopheles gambiae* of the bar-lands zone (*Fst* = 0.2408).

**Conclusion:**

This study revealed that the use of insecticides in Benin for years has altered the genetic structure of *Anopheles gambiae s.s.* populations in all agroecological zones of southern Benin. It would be desirable to orientate vector control efforts towards the use of insecticides other than pyrethroids and DDT or combinations of insecticides with different modes of action.

## Background

Malaria remains a major public health problem worldwide, with an estimated 219 million cases in 2017 [1], most of which (92%) occurred in the WHO African Region [1]. In 2017, 15 countries in sub-Saharan Africa and India have concentrated almost 80% of the total number of malaria cases in the world [1]. *Anopheles gambiae s.s.*, *Anopheles funestus*, and *Anopheles arabiensis* species are the most frequent malaria vectors in sub-Saharan Africa [2]. *Anopheles gambiae s.s.*, the main malaria vector [3], has two molecular forms (M and S) in sub-Saharan Africa [3–5]. However, the recent work of Coetzee et al. [6] suggested these two molecular forms represent distinct species belonging to the *Anopheles gambiae* complex. They are respectively called *Anopheles coluzzii* and *Anopheles gambiae*.

The main control measures against malaria vectors are long-lasting insecticidal mosquito nets (LLINs) and insecticide residual spraying (IRS) [7, 8]. IRS implementation started in Benin in 2008 with the support from President’s Malaria Initiative (PMI). From 2008 to 2010, over 315,000 people out of 350,000 were protected each year by the intervention in the Ouémé Department, southern Benin. In 2011, the operation was relocated from Ouémé to Atacora (northern Benin) where over 650,000 people were protected each year. Due to vector resistance to pyrethroids across Benin, two insecticides from different classes were used in rotation: bendiocarb (a carbamate) and pirimiphos-methyl EC (an organophosphate) [9]. In southern Benin, resistance to pyrethroids is high and LLINs impregnated with deltamethrin or permethrin are the most used vector control tool [10].

The soar of synthetic insecticides began after the Second World War with the discovery in 1939 of the insecticidal properties of dichloro-diphenyl-trichloroethane (DDT) by Müller. Several families of insecticides have been developed over the years. These include pyrethroids, carbamates, organochlorines, and organophosphorus [11, 12]. From the organochlorine family, DDT, a first generation insecticide, has helped reduce or even eradicate malaria in some countries, especially those in Europe [13, 14]. However, its intensive and repeated use has led to the appearance of numerous cases of resistance, therefore limiting its effectiveness [15]. In addition, its high bioaccumulation capacity, its persistence in the environment, and its toxicity in mammals have led to its ban in many countries [16]. Currently, pyrethroids are the insecticides used against mosquitoes in public health and against crop pests [17–19]. Ecologically, Akogbéto et al. [20] reported that several species of mosquitoes, in particular *Anopheles gambiae s.l.*, lay their eggs in cottages located near cultivated areas. These eggs are susceptible to insecticide exposure during crop pest treatments. These same authors have shown that pesticide residues could be found at ground level during metamorphosis of mosquito larvae.

For example, the insecticides or insecticide formulations deltamethrin EC 10.75 g/l, deltamethrin EC 258.75 g/l, endosulfan 350 g/l, and mixture deltamethrin EC 10.75 g/l + triazophos 250 g/l were used in the cotton zones of Benin as well as Orthene, deltamethrin EC 10.75 g/l, and cypermethrin 35 g/l + dimethoate 300 g/l against pests in market gardening areas [20].

Consequently, these chemical agents could exert early on a selective pressure on certain mosquito populations by eliminating susceptible individuals at this stage of development. It is therefore possible that the different agricultural practices implemented have an impact on the resistance status of malaria vectors. Therefore, mosquito resistance to insecticides has become a major public health problem when *Anopheles gambiae* showed resistance to pyrethroids [21]. Several mechanisms allow anopheles to resist the action of insecticides. There are two main mechanisms: resistance related to target modification and metabolic resistance due to overexpression of genes that code for detoxification enzymes [21]. The molecular basis of the insensitivity of active sites of the sodium channel voltage-dependent gene has been characterized in many insect species [22]. From a biochemical point of view, resistance to pyrethroids and DDT in *Anopheles gambiae s.l.* is mainly due to non-synonymous mutations of substitution type at the *L1014* site of the sodium channel voltage-dependent (*Vgsc*) gene [23, 24]. Molecular studies on this same gene have shown other nucleotide mutations on S1–S6 transmembrane segments of domain II [25]. These mutations are responsible for the resistance observed in several groups of species to insecticides [26], translating phylogenetically, an ancestral heritage common to these organisms. This resistance caused by the mutation of the *Kdr* gene is highly developed in *Anopheles gambiae* [27]. In Benin, several previous studies have reported cross-resistance to DDT and pyrethroids in *Anopheles gambiae s.l.* with strong geographical variations along the north-south transect [18, 28]. Other studies have concluded that the *L1014F* mutation in the *Kdr* gene and the overexpression of certain genes in the cytochrome P450 family (Cyp6M2 and Cyp6P3) are potentially involved in mosquito resistance to pyrethroids [29].

There is a body evidence demonstrating that the development of resistance in organisms is a dynamic phenomenon in time and space [30, 31]. Monitoring the evolution of resistance allows a better appreciation of the genetic structure of vector populations in settings subject to intensive use of chemicals associated with other control initiatives. The overarching aim of the present study was to map the distribution of resistance alleles of the *Kdr* gene in malaria vectors in different regions and ecological facies in order to identify the evolutionary forces that might be the basis of anopheline population dynamics.

## Material and methods

### Study zone

The study was conducted in southern Benin from April 2015 to October 2017. Benin is located in West Africa in the tropical zone, more precisely between parallels 6° 30′ and 12° 30′ north latitude, and meridians 1° and 30° 40′ east longitude.

To better study the molecular diversity of *Anopheles gambiae s.s.* species and establish their genetic structure, anopheline populations were defined on the basis of different agroecological zones that take into account pedoclimatic factors and the different cultures practiced [32]. The study mosquitoes were collected in four areas. The central cotton zone or zone 5 is a composite space between the north and the south. Agriculture is practiced by 88% of the population and is usually organized in groups. The bar-lands zone or zone 6 bears its denomination in reference to its type of soil, consisting of sandy clay in a wet state. Crop production bears on maize, groundnuts, cowpea, cassava, yam, taro, chili, coffee, cotton, fruits (mango, citrus, and pineapple), oil palm tree, and vegetable crops. Private irrigation initiatives from artesian drillings or from rivers are being developed for the off-season production of vegetable and rice crops. The fisheries zone or zone 7 includes the coastal sandy strip and the fluvial and lacustrine alluvions of Mono, Ouémé, and Atlantic. Plant production is much diversified but is practiced on relatively small spaces. The zone of the depression or zone 8 extends over the departments of Atlantic, Couffo, Plateau, and Zou. It is particularly characterized by its floristic richness, containing, among others, classified forests, community forests, sacred forests, and palm groves. At the hydrographic level, the Couffo, the Zou, and the Hlan rivers, numerous streams, artesian wells, and shallows offer many agricultural potentialities.

In the bar-lands zone, the districts of Klouékanmè, Dogbo, Toviklin, Avrankou, Porto-Novo, Ifangni, Bohicon, and Zagnanado were chosen. Localities of Bopa, Comé, Lokossa, Cotonou, Sô-Ava, and Adjohoun were chosen in the fisheries zone. We were interested in Djidja and Kétou in the central cotton zone, and finally, Toffo was retained in the zone of the depression.

### Collection of samples

**Fig. 1:**
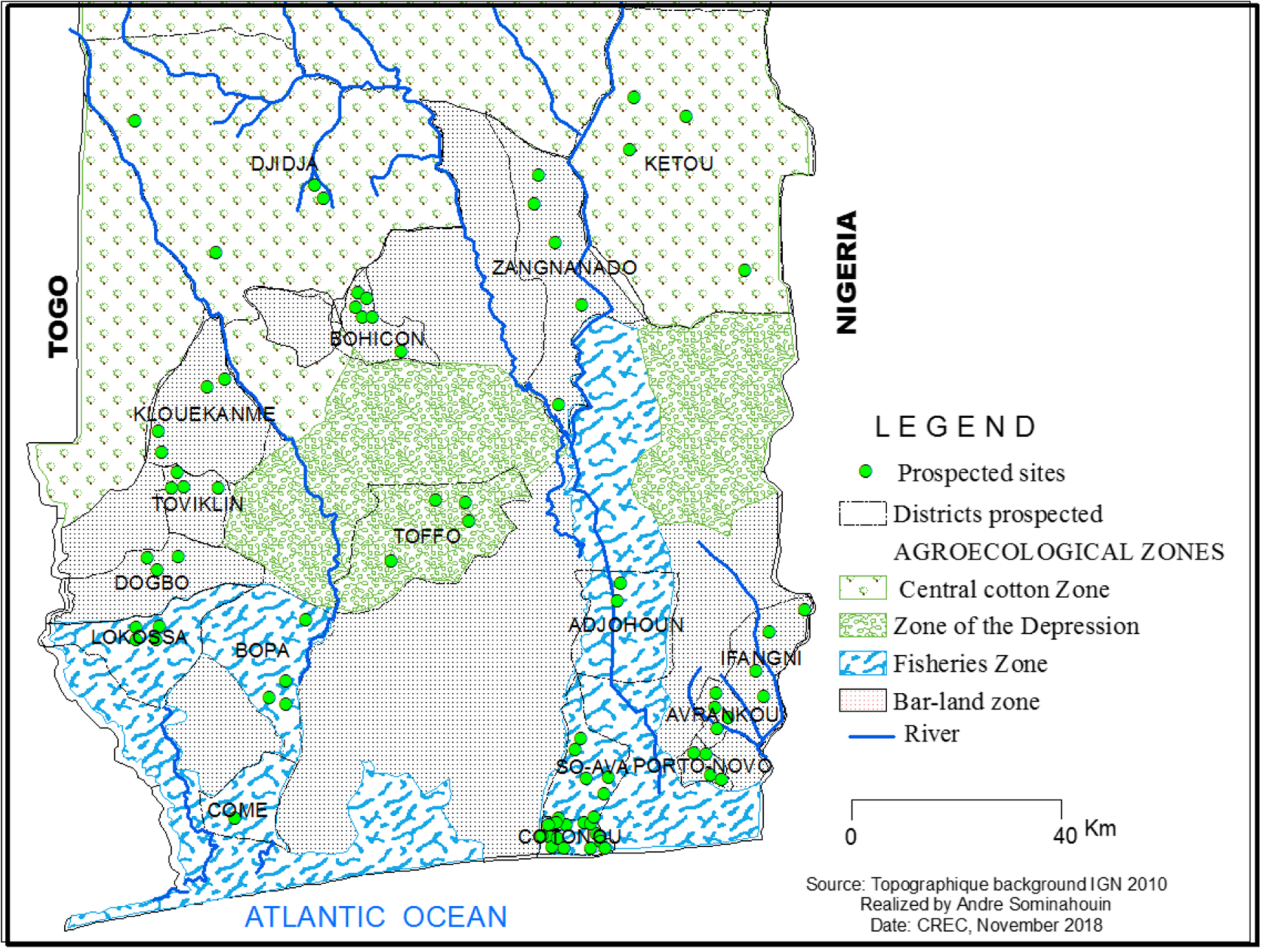
Map showing the distribution of the breeding site surveyed in the different study areas

*Anopheles* larvae were collected in the selected agroecological zones using the “dipping” technique [33]. It consists of taking mosquito larvae from the surface of the breeding site using a ladle. The larvae of *Anopheles gambiae* collected are filtered and then poured into different tanks before being transported to the insectarium of the Entomological Research Center of Cotonou for breeding. The imago obtained after hatching of these eggs is expected to have inherited several common characters from their parents and therefore will be individuals with a very close genetic background. These individuals will subsequently spawn in adulthood in the surrounding breeding site of the same locality causing a high degree of kinship. For this reason, we sampled mosquito larvae at few different sites to minimize the risk of consanguinity and to better establish the genetic structure of different populations. Adults from the emergence of larvae were kept in plastic 1.5-ml tubes (Eppendorf) at − 20 °C for subsequent molecular analyses. Figure 1 shows the sampling sites for this study.

## Molecular characterization of collected samples

### DNA extraction of mosquitoes

Whole mosquitoes were grinded in 200 μl of 2% cetyltrimethylammonium bromide (CTAB). After 5 min of water bath at 65 °C, the blend was mixed with 200 μl of chloroform and then centrifuged at 12,000 rpm for 5 min. The upper portion was gently recovered in another tube and supplemented with 200 μl of isopropanol, homogenized and centrifuged again at 12,000 rpm for 15 min. The liquid in the tube was skillfully spilled so as not to lose the pellet at the bottom. Two hundred microliters of 70% ethanol was added to the pellet for precipitation. After 5 min of centrifugation, the contents of the tube were again finely reversed. The pellet was then drained for at least 3 h on the bench. The extracted DNA was reconstituted with 20 μl of sterile water and left in suspension on the bench all night.

### Identification of different species of *Anopheles gambiae s.l.* collected

The strong morphological similarity between *Anopheles gambiae s.l.* species is often the source of confusion during identification. To overcome this difficulty, it is necessary to supplement morphological studies with molecular analyses. The PCR diagnostic approach used to differentiate between molecular forms is based on the specific and irreversible insertion of a 230 bp transposon (SINE200) on chromosome X of *Anopheles coluzzii* (form M) while this transposon is absent in *Anopheles gambiae* (form S). This genetically hereditary characteristic allows an unambiguous, simple, and direct recognition of molecular forms M and S [34].

**Fig. 2:**
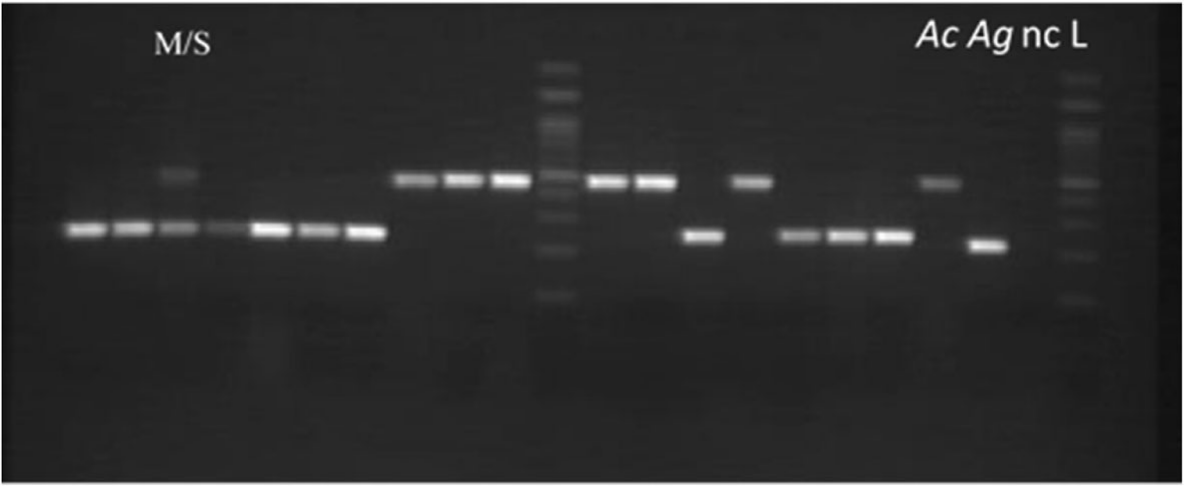
Diagnostic PCR based on *S200* X6.1 primer set in *Anopheles gambiae s.l.* M/S, hybrid form; *Ac, Anopheles coluzzii* (479 bp); *Ag*, *Anopheles gambiae* (249 bp); nc, negative control; L, ladder = 100 bp (Solis BioDyne)

The PCR protocol includes an initial denaturation at 94 °C for 5 min followed by 35 cycles. Each cycle has a denaturation phase at 94 °C for 30 s, primer annealing at 54 °C for 30 s, and elongation by sequential nucleotide additions at 72 °C for 30 s. At the end of the last cycle, a final elongation at 72 °C for 10 min is carried out to allow complete amplification of the sequences. In addition to the conventional components of PCR, the following primers were used: 200X6.1F-TCGCCTTAGACCTTGCGTTA and200X6.1R-CGCTTCAAGAATTCGAGATAC [34]. Amplified DNA copies are stored at a final temperature of 4 °C prior to migration on a 1.5% agarose gel with ethidium bromide used as intercalating agent and migration front. Figure 2 illustrates the electrophoretic profile of each species obtained from our experiments in the laboratory. The literature clearly states that *Anopheles quadriannulatus* is not found in Benin while *Anopheles melas* is ecological to brackish water. As we did not sample in lagoon/coastal environments, all mosquitoes were subjected to PCR using the protocol of Santolamazza et al. (2008). All mosquitoes were subjected to PCR using the protocol of Scott et al. [35] to confirm again the absence of other members of the *Anopheles gambiae* complex. The protocol consists in determining the polymorphisms in the intergenic spacer (IGS) of ribosomal DNA. It allows the identification of the different sibling species of the *Anopheles gambiae* complex namely *Anopheles gambiae s.s*, *Anopheles arabiensis*, *Anopheles melas*, *Anopheles merus*, *Anopheles quadriannulatus*, and *Anopheles bwambae.* For that, the following primers were used:

- UN: GTGTGCCGCTTCCTCGATGT
- AG: CTGGTTTGGTCGGCACGTTT
- AA: AAGTGTCCTTCTCCATCCTA
- ME: TGACCAACCCACTCCCTTGA
- QD: CAGACCAAGATGGTTAGTAT

The UN primer anneals to the same position of the rDNA of all five species, and AG anneals specifically to *Anopheles gambiae*. ME anneals to both *Anopheles merus* and *Anopheles melas.* AA anneals to *Anopheles arabiensis* and QD anneals to *Anopheles quadriannulatus.*

The PCR was carried out using a program of 30 cycles of denaturation at 94 °C for 30 s, annealing at 50 °C for 30 s, and extension at 72 °C for 30 s. Amplified DNA copies are stored at a final temperature of 4 °C prior to migration on a 2.5% agarose gel with ethidium bromide used as intercalating agent and migration front.

### Detection of the *L1014F* mutation of the *Kdr* gene

The presence of resistance alleles (*L1014F*) of the *Kdr* gene in samples taken at each study site was detected by PCR following the protocol described by Martinez-Torres et al. [24]. Indeed, PCR-PASA consists of using the specific primers called Agd1, Agd2, Agd3, Agd4, and the Taq polymerase to search by amplification for resistant or susceptible alleles on the fragment coding for *Vgsc*. The Agd1/Agd2 primer pair flanks the *Kdr* gene amplifying a 293 bp product as a control. The Agd3/Agd1 primer pairs only with the *Kdr* gene resistance allele to amplify a 195pb fragment. Finally, the Agd4/Agd2 primer pair associates only with the gene’s sensitive allele amplifying a 137pb fragment. The nucleotide sequences of these primers are as follows [24]:

- Agd1: 5′-ATAGATTCCCCGACCATG-3′;
- Agd2: 5′-AGACAAGGATGATGAACC-3′;
- Agd3: 5′-AATTTGCATTACTTACGACA-3′;
- Agd4: 5′-CTGTAGTGATAGGAAATTTA-3′.

### Analysis of the genetic structure of the studied species

Several analytical approaches were used to establish the genetic structure of populations. The genetic constitution of each population was determined by calculating the allelic and genotypic frequencies of the *Kdr* gene. Genepop software version 4.2 was used to calculate these frequencies. The *p* value associated with each gene frequency was calculated with the binomial test function in R software version 3.3.3. The Hardy-Weinberg equilibrium test (HWE) using the Genetics software version 1.3.8.1 allowed to check whether there was panmixia in the *Anopheles* populations of the agroecological zones. Other indices to identify the probable causes of a possible deviation from panmixia were calculated using the Weir & Cockerham (W&C) [36] and Robertson & Hill [37] formulas included in the same Genepop software version 4.2. These indices include observed heterozygosity (*Ho*), expected heterozygosity (*He*), fixation index (*Fis*), and within populations (*Fst*) and between populations (*Fsc*) genetic differentiations. The fixation index (*Fis*) allows to quantify the difference to panmixia. If *Fis* < 0, the population has an excess of heterozygote, but if *Fis* > 0, it has a heterozygote deficiency. The criteria used to assess genetic differentiation within populations are those defined by Hartl [38]. Genetic differentiation is low if the *Fst* value is less than or equal to 0.05, moderate when its value is between [0.05; 0.15], and large when it is between [0.15; 0.25]. This differentiation is very large if the *Fst* value is greater than 0.25.

## Results

### Molecular identification of the different species collected

Table 1 presents the different species of the *Anopheles gambiae* complex identified. In all agroecological zones, *Anopheles coluzzii* was the major species. The analysis of the results revealed that whatever is the agroecological zone, *Anopheles coluzzii* was the major species. The proportion of *Anopheles gambiae* was low in most agroecological zones except for the bar-lands zone. In the depression zone, *Anopheles coluzzii* accounted for 75% of the species while the proportion of *Anopheles gambiae* was 25%. No hybrid (*Anopheles coluzzii*/*Anopheles coluzzii*) was found in this zone. In the bar-lands zone, 59.64% of *Anopheles coluzzii* were found against 38.91% for *Anopheles gambiae*. Hybrids accounted for only 1.45% in this zone. Considering the cotton zone of the center, we obtained 82.86% of *Anopheles coluzzii* and 17.14% of *Anopheles gambiae*. In the fishery zone, data revealed 93.89% of *Anopheles coluzzii*, 5.28% of *Anopheles gambiae*, and 0.83% of hybrids. Careful analysis considering the sampled localities revealed that, exceptionally in Bohicon and Klouékanmè which belongs to the bar-lands zone, *Anopheles gambiae* was in majority. Only a few hybrid forms have been found in some localities (Cotonou, Porto-Novo, Ifangni).

**Table 1:** Molecular identification of species of the *Anopheles gambiae* complex collected from the different agroecological zones

Agroecological zones	Localities	Period of larvae collection	Geographic coordinates		Number tested	*Anopheles coluzzii*	*Anopheles gambiae*	Hybrid forms
			Latitude	Longitude				
Zone of the depression	Toffo	October 2017	6° 56′ 20.73″ N	2° 04′ 59.05″ E	28	21	7	0
	Total				28	21	7	0
Bar-lands zone	Klouékanmè	June 2016	7° 03′ 08.61″ N	1° 51′ 57.42″ E	32	3	29	0
	Dogbo	June 2016	6° 48′ 19.18″ N	1° 47′ 16.53″ E	39	27	12	0
	Toviklin	June 2016	6° 56′ 20.73″ N	1° 46′ 24.08″ E	25	21	4	0
	Avrankou	June 2017	6° 33′ 42.31″ N	2° 38′ 55.35″ E	15	15	0	0
	Porto-Novo	April 2015	6° 29′ 53.71″ N	2° 37′ 42.60″ E	54	47	4	3
	Ifangni	September 2016	6° 40′ 44.49″ N	2° 43′ 02.62″ E	42	28	13	1
	Bohicon	May 2016	7° 10′ 48.42″ N	2° 04′ 17.44″ E	39	4	35	0
	Zagnanado	September 2017	7° 16′ 49.94″ N	2° 24′ 29.25″ E	29	19	10	0
	Total				275	164	107	4
Central cotton zone	Djidja	September 2017	7° 20′ 46.64″ N	1° 56′ 08.00″ E	29	27	2	0
	Kétou	April 2016	7° 21′ 37.44″ N	2° 36′ 14.24″ E	41	31	10	0
	Total				70	58	12	0
Fisheries zone	Bopa	September 2016	6° 42′ 12.40″ N	1° 56′ 56.48″ E	30	30	0	0
	Comé	September 2016	6° 24′ 34.78″ N	1° 53′ 07.35″ E	32	26	6	0
	Lokossa	September 2016	6° 38′ 43.20″ N	1° 43′ 11.36″ E	25	18	7	0
	Cotonou	April 2015/June 2017	6° 22′ 16.82″ N	2° 23′ 29.71″ E	175	167	5	3
	Sô-Ava	June 2017	6° 28′ 07.75″ N	2° 24′ 06.53″ E	80	80	0	0
	Adjohoun	April 2017	6° 42′ 09.74″ N	2° 29′ 56.18″ E	18	17	1	0
	Total				360	338	19	3

### Genotyping of mosquitoes and genetic structure of populations

#### Calculations of genotypic and allelic frequencies in the different agroecological zones and test of Hardy-Weinberg equilibrium

The *L1014F* mutation of the *Kdr* gene was analyzed in the different species identified in all agroecological zones. The genetic structure results are summarized in Table 2. The resistant homozygous individuals (*1014F*/*1014F*) were mostly represented in all agroecological zones. In *Anopheles gambiae*, the frequency of resistant homozygous individuals was 85.71% in the zone of the depression, 92.52% in the bar-lands zone, 83.33% in the central cotton zone, and 100% in the fisheries zone. Heterozygous individuals of genotype *1014 L*/*1014F* were scarcely represented including 14.29% in the zone of the depression, 4.67% in the bar-lands zone, 16.67% in the central cotton zone, and none in the fisheries zone. Susceptible homozygote individuals (*1014 L*/*1014 L*) were only found in the bar-lands zone with a frequency of 2.81%. The frequency of the *L1014F* resistant allele was strongly represented *Anopheles gambiae* populations with a mean frequency exceeding 90% across all four agroecological zones. The HWE was respected in the *Anopheles gambiae* populations of the zone of the depression and of the central cotton zone (*p* value > 0.05). This equilibrium was not observed in the populations of the bar-lands zone (*p* value < 0.05). A strong selection of resistant homozygote individuals was recorded in the fisheries zone.

**Table 2:** Genotypic and allelic frequencies and HWE test of *Anopheles gambiae* and *Anopheles coluzzii* populations

Species	Agroecological zones	Numbers	Genotypes (genotypic frequencies %)			Frequency %	*p* value	*p* value
			*1014F*/*1014F*	*1014 L*/*1014F*	*1014 L*/*1014 L*	(*1014F*)	*F* (*1014F*)	(*H-W*)
*Anopheles gambiae*	Zone of the depression	7	6 (85.714)	1 (14.286)	0 (0.000)	92.857	0.0018	1
	Bar-lands zone	107	99 (92.523)	5 (4.673)	3 (2.804)	93.548	< 0.0001	0.0007
	Central cotton zone	12	10 (83.333)	2 (16.667)	0 (0.000)	91.666	< 0.0001	1
	Fisheries zone	19	19 (100.000)	0 (0.000)	0 (0.000)	100.000	< 0.0001	–
	Total	145	134 (92.414)	8 (5.517)	3 (2.069)	95.172	< 0.0001	0.0017
*Anopheles coluzzii*	Zone of the depression	21	18 (85.714)	1 (4.762)	2 (9.524)	88.095	< 0.0001	0.0094
	Bar-lands zone	164	128 (78.049)	25 (15.244)	11 (6.707)	85.671	< 0.0001	< 0.0001
	Central cotton zone	58	48 (82.759)	7 (12.069)	3 (5.172)	88.793	< 0.0001	0.0153
	Fisheries zone	338	269 (79.586)	57 (16.864)	12 (3.550)	88.018	< 0.0001	0.0008
	Total	581	463 (79.690)	90 (15.411)	28 (4.819)	87.435	< 0.0001	< 0.0001

*1014F/1014F* homozygote resistant, *1014 L/1014F* heterozygote, *1014 L/1014 L* homozygote susceptible, *F (1014F)* frequency of resistance allele, *p value (1014F)* threshold of significance of the frequency, *p value (HWE) p* value to the Hardy-Weinberg equilibrium

In *Anopheles coluzzii* species, resistant homozygote individuals (*1014F*/*1014F*) exhibited relatively high frequencies in the different agroecological zones studied. In the zone of the depression, 85.72% of individuals had this genotype. This frequency was 78.05% in the bar-lands zone, 82.76% in the central cotton zone, and 79.59% in the fisheries zone. Heterozygous individuals (*1014 L*/*1014F*) showed variable frequencies depending on the agroecological zone. There were 4.762% heterozygous individuals in the zone of the depression, 15.24% in the bar-lands zone, 12.07% in the central cotton zone, and 16.86% in the fisheries zone. Homozygote susceptible individuals (*1014 L/1014 L*) exhibited low frequencies in all agroecological zones. There were 9.52% in the zone of the depression, 6.71% in the bar-lands zone, 5.17% in the central cotton zone, and 3.55% in the fisheries zone. All *Anopheles coluzzii* populations in the study areas showed high frequencies for the resistance allele *1014F*. It was 88.09% in the zone of the depression, 85.67% in the bar-lands zone, 88.79% in the central cotton zone, and 88.018% in the fisheries zone. None of these populations respected the Hardy-Weinberg equilibrium (*p* value < 0.05).

### Panmixia gap

Table 3 presents the heterozygosity observed (*Ho*), the heterozygosity expected (*He*), and the fixation index (*Fis*) in *Anopheles coluzzii* and *Anopheles gambiae* across the different agroecological zones. Heterozygosity observed was lower than expected heterozygosity in all *Anopheles coluzzii* populations, suggesting a deficit of heterozygosity (*Fis* > 0). Similarly, this heterozygosity deficit was observed in *Anopheles gambiae* populations collected from the bar-lands zone. An excess of heterozygosity (*Fis* < 0) was observed in *Anopheles gambiae* populations collected from the central cotton zone. However, in the few populations where panmixia was observed, heterozygosity was balanced: there was neither deficit nor excess.

**Table 3:** Quantification of panmixia gap in *Anopheles coluzzii* and *Anopheles gambiae*

Population	*Anopheles coluzzii*			*Anopheles gambiae*		
	*Ho*	*He*	*Fis* (W&C)	*Ho*	*He*	*Fis* (W&C)
Zone of the depression	0.048	0.215	0.782	0.143	0.143	0.000
Bar-lands zone	0.152	0.246	0.382	0.047	0.098	0.542
Central cotton zone	0.120	0.189	0.401	0.105	0.100	− 0.048
Fisheries zone	0.169	0.210	0.202	0	0.000	–
Total	0.155	0.219	0.296	0.055	0.092	0.000

*Ho* observed heterozygosity, *He* expected heterozygosity, *Fis* (W&C) fixation index (Weir & Cockerham)

### Genetic differentiation between populations

Genetic differentiations were calculated within and between different *Anopheles* populations for both *Anopheles coluzzii* and *Anopheles gambiae* (Table 4). For both species, no genetic differentiation was observed between populations (*Fsc = 0*). In *Anopheles coluzzii*, low genetic differentiation was observed in the bar-lands and the central cotton zones (*Fs*t < 0.05), while a moderate differentiation was observed in the fisheries zone (*Fst* = 0.13). In *Anopheles gambiae*, the genetic differentiation was very large (*Fst* = 0.2408) in the bar-lands zone.

**Table 4:** Genetic differentiation of *Anopheles coluzzii* and *Anopheles gambiae* populations

Agroecological zone	*Anopheles coluzzii*		*Anopheles gambiae*	
	*Fst* (W&C)	*Fsc* (W&C)	*Fst* (W&C)	*Fsc* (W&C)
Zone of the depression	–	0.000	–	0.000
Bar-lands zone	0.0077		0.2408	
Central cotton zone	0.0313		–	
Fisheries zone	0.1295		–	

*Fst (W&C)* genetic differentiation within populations, *Fsc(W&C)* genetic differentiation between population

## Discussion

The molecular identification of the different species revealed that *Anopheles coluzzii* and *Anopheles gambiae* are represented in all agroecological zones. Several studies have shown [39, 40] that these two species are the most widespread in sub-Saharan Africa. Each species seems to have an ecological preference because of its geographical distribution and its biology. As a result, the preferred zones of both species differ. *Anopheles coluzzii* is mostly found in fisheries zones where availability of water favors the development of permanent breeding sites. Studies in Ghana have shown that *Anopheles coluzzii* prefers to lay eggs in permanent settlements [41, 42]. The particularities observed at Bohicon and Klouékanmè in the bar-lands zone where *Anopheles gambiae* occurs in large numbers can be explained by the sampling period characterized by the existence of favorable ecological conditions.

Hybrid forms were only observed in three localities of the studied agroecological zones (Cotonou, Porto-Novo, Ifangni). These results confirmed that *Anopheles coluzzii* and *Anopheles gambiae* are distinct species whose biological separation is not nearly complete. Coetzee et al. [6] have shown that *Anopheles gambiae* and *Anopheles coluzzii* are two distinct species. Nevertheless, in the agroecological zones studied, *Anopheles coluzzii* and *Anopheles gambiae* live in sympatry. This remark reflects an adaptation of these two species to different breeding sites and ecology. Several studies have proved the sympatric life between the different species of *Anopheles gambiae* complex and even with other kinds of mosquitoes [42–44].

The resistant allele of the *Kdr* gene is strongly selected in all agroecological zones. The high allelic frequency observed in these zones has several origins including farming practices that require extensive use of several classes of pesticides to effectively control insect pests [28], widespread use of LLINs, indoor residual spraying (IRS) [45], and many other factors that are yet to be elucidated. However, insecticides of the pyrethroid family are also used to impregnate mosquito nets. The products available to public health for mosquito control are the same as those used for decades in agriculture against pests. This massive and continuous use of pyrethroids in public health and agriculture could also explain the strong selection of the resistance allele in these two species in the different agroecological zones. Akogbéto et al. [20] showed that pesticide residues can exert a selective pressure on certain mosquito populations as early as the larval life by eliminating sensitive individuals at this stage of development. For example, in Cotonou, a locality in the fisheries zone, with the exception of LLINs, another source of selection pressure is the use of pesticides for market gardening in Houéyiho [28] and other sites in the city. The constant availability of breeding sites due to the hydromorphic nature of the soil in this locality could be at the origin of a rapid expansion of resistant individuals from marker gardening sites throughout Cotonou.

The genetic structure of most of the anopheline populations analyzed is panmixia except for the central cotton zone and the zone of the depression. It has been shown in population genetics that the causes of such a Hardy-Weinberg equilibrium shift may be the Wahlund effect, consanguinity, genetic drift, selection against heterozygotes, or their combination. Based on cultural practices in Benin, one is tempted to think of a selection of the resistant allele in a high insecticide-use environment, even if the other factors are not to be neglected. The observed HWE in the abovementioned zones may be related to the relatively small numbers of mosquitoes used for data analysis, sampling period or punctual migration of individuals favoring gene flow, and random crosses between individuals. The heterozygote deficiency is widely observed within all populations. This observation can be translated by the effect of insecticides on mosquitoes that would eliminate susceptible individuals in different populations and leaving the first resistant, which in turn through preferential mating and consanguinity will transmit almost exclusively the allele of resistance to their descendants and consequently ensure the expansion of this genetic mutation within populations. No genetic differentiation was observed between the different agroecological zones regardless of the species considered. The lack of genetic differentiation observed between the different agroecological zones is linked to the strong selection of resistant individuals in these areas and, to some extent, the resolving power of the type of marker used.

Nevertheless, within each agroecological zone, some genetic differentiations were observed. Genetic differentiation was low in the central cotton zone and the zone of the depression in *Anopheles coluzzii*. This phenomenon is due to an apparent homogeneity of biotic and abiotic factors that can create genetic differentiation between individuals within this population. The moderate genetic differentiation observed in the fisheries zone could be due to a variability of various factors capable of exerting insecticidal pressures and the effect of human actions on *Anopheles* populations in the different localities of this zone. The permanent availability of breeding sites could also reduce the phenomenon of migration in this region by promoting a certain genetic structuring of *Anopheles* populations. In the fisheries zone, the presence of market gardening sites in Cotonou coupled with a strong domestic use of pesticides contributes to a strong selection of the resistance allele [29]. The variation of the selection pressures observed in different localities of this agroecological zone is at the origin of the moderate differentiation observed in *Anopheles* populations. The high genetic differentiation observed in *Anopheles gambiae* in the bar-lands zone is believed to be related to the highly heterogeneous geographical distribution of this species.

The *Anopheles* collected from the bar-lands zone must be adapted to the ecology of their environment. The variability of selection pressures associated with the low representativeness of *Anopheles gambiae* in the different localities of this zone could partly explain the great genetic differentiation observed. The generalized heterozygote deficit observed in the *Anopheles* populations reflects the existence of a characteristic consanguinity within each species. It would be desirable to deepen this study using more advanced methodological and analytical approaches such as sequencing to better assess genetic differentiation between different populations.

The study of vectors’ population genetic structure is required before the implementation of any insecticides or chemicals based control intervention. Indeed, the selection of an individual resistance allele is often due to the large use of chemicals over the years [46, 47]. An effective control of vectors and other undesirable organisms calls for good knowledge of their diversity and genetic structure in order to identify probable causes that could lead to the panmixia gap. This will help in the choice of adequate control method. Although the resolving power of our molecular marker remains questionable, the present results lead us to believe that consanguinity and overuse of insecticides are factors that would favor the selection of the resistant allele L1014F in populations of *Anopheles gambiae s.s.* in Benin.

## Conclusions

This study established the genetic structure of *Anopheles gambiae* and *Anopheles coluzzii* species using the *Kdr* gene in four agroecological zones of Benin. The resistance allele was strongly selected in almost all agroecological zones for both species. A panmixia gap was observed in all agroecological zones studied with a heterozygosity deficit in most populations. The low within and between individual genetic differentiations reflected the strong selection of the resistant allele in these *Anopheles* populations. It would be desirable to further study the molecular diversity of malaria vectors by using more resolute markers to better establish the fine genetic structure of their populations.

## Abbreviations

CTAB: Cetyltrimethylammonium bromide
DNA: Deoxyribonucleic acid
*Fis*: Fixation index
*Fsc*: Genetic differentiations between populations
*Fst*: Genetic differentiations within populations
*He*: Expected heterozygosity
*Ho*: Observed heterozygosity
HWE: Hardy-Weinberg equilibrium
IRS: Indoor residual spraying
*Kdr*: Knockdown resistance
LLIN: Long-lasting insecticidal nets
PCR: Polymerase chain reaction
W&C: Weir & Cockerham
WHO: World Health Organization
